# Forecasting Emergency Room Patient Volumes Using Extreme Gradient Boosting With Temporal and Seasonal Feature Engineering: A Comparative Study Across Hospitals

**DOI:** 10.7759/cureus.86276

**Published:** 2025-06-18

**Authors:** Kian A Huang, William M Hardin, Neelesh S Prakash

**Affiliations:** 1 Radiology, University of South Florida Morsani College of Medicine, Tampa, USA

**Keywords:** ai and machine learning, ed overcrowding, emergency medicine research, predictive healthcare analytics, predictive modeling

## Abstract

Objective: Accurate forecasting of emergency department (ED) patient volumes is critical for optimizing hospital resource allocation and staffing. This preliminary study evaluates the performance of an eXtreme Gradient Boosting (XGBoost)-based regression model in predicting daily ED visit counts across three simulated hospitals, using time-series features derived from synthetic hospital data retrieved from a publicly available Kaggle dataset (n=300).

Methods: For each hospital, we trained an XGBoost model using engineered temporal features, recent lagged values, and rolling averages of past patient volumes. Feature engineering included day of the week, month, week of the year, quarter of the year, and weekend status. Model performance was benchmarked against three general baselines: a naive lag-1 predictor, a constant mean predictor, and a three-day rolling mean. Performance was assessed using mean squared error (MSE), root MSE (RMSE), mean absolute error (MAE), and R² score.

Results: The XGBoost model consistently outperformed all baseline methods across all hospitals. For Hospital 101, it achieved an R² of 0.55 compared to 0.27 for the rolling mean and negative R² values for naive and mean baselines. Hospital 102 showed improved accuracy with an R² of 0.69 versus 0.12 for the rolling mean. The best performance was observed at Hospital 103, where XGBoost achieved an R² of 0.81, significantly outperforming all baselines. Across all sites, XGBoost reduced RMSE and MAE by more than 40% relative to the best-performing baseline.

Conclusion: Leveraging temporal and historical patterns in simulated ED data, the XGBoost model delivers markedly more accurate volume forecasts than traditional baseline methods. These findings on synthetic data support the potential for machine learning-based forecasting models in enhancing hospital operational decision-making, with future directions involving the use of real-world hospital data.

## Introduction

Emergency department (ED) overcrowding is a pervasive global issue impacting patient care and hospital operations. Overcrowding leads to increased patient waiting times, delays in treatment, and higher rates of patients leaving without being seen, which can result in worsening health conditions and subsequent hospitalizations [1]. Studies have shown that the quality of treatment deteriorates in overcrowded situations, with notable delays in test and imaging results, consultations, and overall patient management [1].

ED overcrowding has been consistently associated with negative outcomes across various healthcare systems. A Finnish retrospective observational study found that non-critical patients admitted during periods of high ED crowding experienced a statistically significant increase in 10-day mortality, underscoring that even patients not in immediate danger are adversely affected by congestion [2]. Similarly, an Australian study reported a clear rise in mortality among patients admitted during overcrowded shifts compared to normal periods [3]. The strain caused by overcrowding also impacts healthcare staff, contributing to decreased job satisfaction and increased burnout, which in turn reduces available personnel and further exacerbates system inefficiencies [1]. Financially, overcrowding leads to higher treatment costs due to more frequent reconsultations, increased hospital admissions, and longer lengths of stay [1]. Together, these findings highlight the urgent need for systemic solutions to ED crowding to improve patient outcomes, support healthcare workers, and reduce economic burdens.

Given these multifaceted consequences of ED overcrowding, proactive strategies are essential. One such approach is the use of short-term forecasting models, which allow hospitals to anticipate patient surges and allocate resources accordingly, thereby mitigating the operational and clinical burdens of overcrowding. Accurate short-term forecasting of ED visits is crucial for effective resource allocation and improving patient care. With the rising demand for emergency services, hospitals face challenges in maintaining quality care and operational efficiency. Implementing predictive models that utilize calendar or temporal variables, such as the day of the week, month, week of the year, quarter of the year, and weekend status, has been shown to enhance forecasting accuracy [4]. For example, a study analyzing daily ED visit data found that incorporating temporal features - such as the day of the week - significantly enhanced the accuracy of patient volume forecasts. The study identified consistent weekly patterns, with the highest volumes on Mondays and the lowest on weekends, which supported more effective staffing and resource planning [4]. By leveraging such predictive models, hospitals can better anticipate patient surges, optimize scheduling, and mitigate the negative impacts of overcrowding. Another study by Schweigler et al. [5] evaluated the effectiveness of traditional historical average models compared to more sophisticated time series methods such as seasonal autoregressive integrated moving average (ARIMA) models. The research demonstrated that ARIMA models significantly outperformed historical averages in forecasting ED bed occupancy for 4- and 12-hour horizons across multiple hospitals [5]. This indicates that traditional methods may not adequately account for the dynamic and seasonal fluctuations in ED demand. Similarly, a study published in the British Journal of Healthcare Management highlighted the limitations of relying solely on historical trends and averages for predicting ED arrivals, emphasizing that such methods often fail to consider external factors such as seasonality and randomness, leading to less accurate forecasts [6]. Furthermore, a comprehensive review by Gul et al. [7] analyzed various statistical forecasting models applied in ED settings. The review found that, while traditional methods such as linear regression and ARIMA models are useful, they often struggle with the non-linear and random nature of ED demand [7]. The authors suggested that more complex models, combining different forecasting techniques, could offer better accuracy in capturing the complexities of ED utilization patterns. These findings underscore the need for more advanced forecasting techniques to enhance the accuracy of ED arrival predictions.

To address these limitations, this study introduces eXtreme Gradient Boosting (XGBoost), a high-performance, tree-based ensemble learning algorithm that has gained widespread use for its speed, scalability, and ability to model complex, non-linear relationships [8]. Unlike traditional statistical models, XGBoost can effectively handle large datasets with intricate feature interactions and missing values, making it well-suited for forecasting dynamic healthcare demand. In the context of emergency care, XGBoost has shown promise in producing more accurate and robust predictions, particularly when enriched with engineered temporal features, such as day of the week, month, and holiday indicators [8]. This preliminary methodological study aims to assess the effectiveness of an XGBoost-centered predictive model in capturing hospital-specific ED visit patterns and comparing its performance against baseline statistics.

## Materials and methods

Dataset

We utilized a simulated ED dataset obtained from Kaggle, structured in a comma-separated values (CSV) format (n=300). The data include three main columns: hospital_id (specifying one of three unique hospital identification numbers: 101, 102, or 103), date, and patient_count (total daily patient volume for a given date). Each row represents the daily number of ED visits for a specific hospital on a particular date. The range of dates used per hospital was 2024-01-01 to 2024-04-09 (year-month-day). The dataset was preprocessed by first converting the date column to a datetime format. Data were then grouped by hospital_id and date. Missing values resulting from feature engineering operations, such as shifting or rolling averages, were removed using “dropna()” to ensure model stability. All data preprocessing, modeling, and evaluation were conducted using Python 3.10, with key libraries, including pandas (v1.5.3), scikit-learn (v1.2.2), XGBoost (v1.7.6), and SHAP (v0.41.0).

Feature engineering

To capture temporal dynamics, we extracted the day of the week (0-6) and month (1-12) from each date, reflecting weekly and seasonal patterns in ED utilization [4]. We created lag features for the previous seven days (the values from the past seven days with respect to a current predictive timepoint) to enable the model to learn from recent trends. Additionally, a three-day rolling mean was computed to smooth out short-term fluctuations while maintaining responsiveness. The choice of a seven-day lag window aligns with weekly behavioral cycles in hospital admissions, while a three-day rolling mean was selected to balance noise reduction with responsiveness to recent changes [9]. Public holiday and calendar anomaly indicators were not included due to their absence in the synthetic dataset. In future applications using real-world data, these features could provide valuable signals for modeling atypical surges or lulls in ED volume.

Model development

Three models were trained for each hospital using the XGBoost regressor, a tree-based ensemble method optimized for speed and accuracy [8]. Key hyperparameters included n_estimators=500, learning_rate=0.05, max_depth=6, and early_stopping_rounds=20, max_depth=6, subsample=0.9, and colsample_bytree=0.9. To preserve the temporal nature of the data, we employed a non-shuffled split into training (64%), validation (16%), and test (20%) sets, maintaining chronological order. A fixed random seed (random_state=42) was applied during dataset splitting and model initialization to ensure reproducibility of results across runs. Features and targets were normalized using “MinMaxScaler” (a data normalization technique to limit a range of data from 0 to 1) to improve training convergence and performance, especially important due to the wide range in patient volumes across hospitals. Although XGBoost does not require feature scaling, MinMaxScaler was applied primarily to the target variable (patient_count) to facilitate convergence during model training and to improve loss sensitivity across different hospital scales. Input features were left unscaled unless explicitly numeric (e.g., rolling averages).

The selected hyperparameters (e.g., n_estimators=300, learning_rate=0.05) were based on performance in prior studies involving time series regression tasks. While no formal grid search or cross-validation was performed, parameters were manually tuned using the validation set to balance bias and variance while ensuring training efficiency. The validation set was used for early stopping, with a patience parameter of 20 rounds. Model performance on the validation set was monitored to prevent overfitting, and training ceased once the loss failed to improve within the specified rounds.

Baseline comparisons

We implemented three baseline forecasting strategies for comparison: naive forecast (uses the value of lag_1, or the previous day, as the prediction), rolling mean (predicts using the three-day rolling average), and the mean baseline, which is a constant value equal to the mean of the training data of hospital dates. These baselines provide context for evaluating the added value of the XGBoost model. All baseline models were evaluated using the same chronological train-validation-test split as the XGBoost model to ensure consistency and fairness in comparative analysis.

Evaluation metrics

To assess the performance of the predictive model, four key evaluation metrics were utilized: mean squared error (MSE), root MSE (RMSE), mean absolute error (MAE), and the coefficient of determination (R²). The MSE measures the average squared difference between the predicted and actual values, penalizing larger errors more severely and providing a sense of overall prediction accuracy. The RMSE, derived as the square root of MSE, expresses this error in the same unit as the target variable, making it more interpretable in practical terms. The MAE calculates the average absolute difference between predictions and actual values, offering a straightforward indication of prediction accuracy without emphasizing larger errors. Finally, the R² score indicates the proportion of variance in the dependent variable that is predictable from the independent variables, with values closer to 1.0 suggesting stronger model performance. To ensure fair and meaningful comparison, these metrics were computed separately for each hospital, recognizing potential differences in patient volumes, seasonal patterns, and trend behaviors across facilities.

Model interpretability

To enhance transparency, we applied a SHapley Additive exPlanations (SHAP) figure for each model to interpret the influence of each input feature on the model’s predictions. SHAP summary plots were generated to visualize the global importance and directionality of each feature, aiding clinical relevance and trust. SHAP values were computed using TreeExplainer from the SHAP library, applied to the test set predictions to assess the global feature importance and interpretability of the final XGBoost models.

## Results

Predictive performance across hospitals

The XGBoost model outperformed all baseline approaches across all three hospitals, demonstrating strong generalization and predictive accuracy, as shown in Table 1. For Hospital ID 101, the XGBoost model achieved an MSE of 35.84, RMSE of 5.99, MAE of 5.17, and an R² of 0.55, substantially improving over the naive baseline (R² = -1.15) and rolling mean (R² = 0.27). In Hospital ID 102, XGBoost yielded even stronger results, with MSE of 25.39, RMSE of 5.04, MAE of 4.09, and an R² of 0.69, significantly exceeding the performance of the rolling mean baseline (R² = 0.12). For Hospital ID 103, the best results were achieved, with an MSE of 8.15, RMSE of 2.86, MAE of 2.32, and an R² of 0.81, indicating a high degree of precision in short-term patient volume forecasting. This reflects the model’s ability to adapt to smoother or more predictable patient flow patterns when present.

**Table 1:** Summary of the comparative performance across all metrics across all hospital sites Hospital ID = Identification number of each hospital; XGBoost = Extreme gradient boosting; MSE = Mean squared error; RMSE = Root mean squared error; MAE = Mean absolute error; R² = Coefficient of determination

Hospital ID	Model	MSE	RMSE	MAE	R²
101	XGBoost	35.84	5.99	5.17	0.55
101	Naive	169.89	13.03	10.53	-1.15
101	Mean	79.26	8.9	7.89	0
101	Rolling Mean	57.71	7.6	6.28	0.27
102	XGBoost	25.39	5.04	4.09	0.69
102	Naive	212.42	14.57	12.95	-1.57
102	Mean	87.42	9.35	8.26	-0.06
102	Rolling Mean	72.44	8.51	6.95	0.12
103	XGBoost	8.15	2.86	2.32	0.81
103	Naive	67.84	8.24	6.47	-0.58
103	Mean	43.26	6.58	5.79	-0.01
103	Rolling Mean	27.42	5.24	4.25	0.36

Forecast visualization

**Figure 1:**
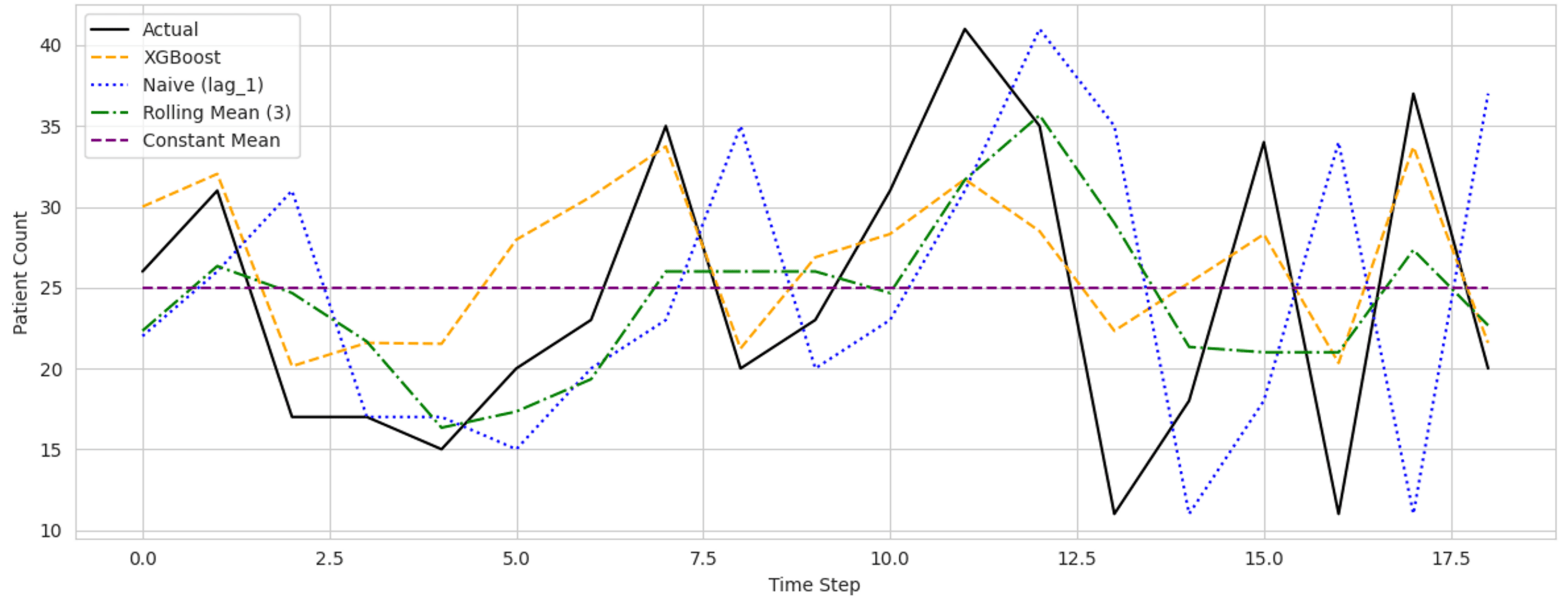
Predicted versus actual daily patient counts across Hospital ID 101 XGBoost = Extreme gradient boosting

**Figure 2:**
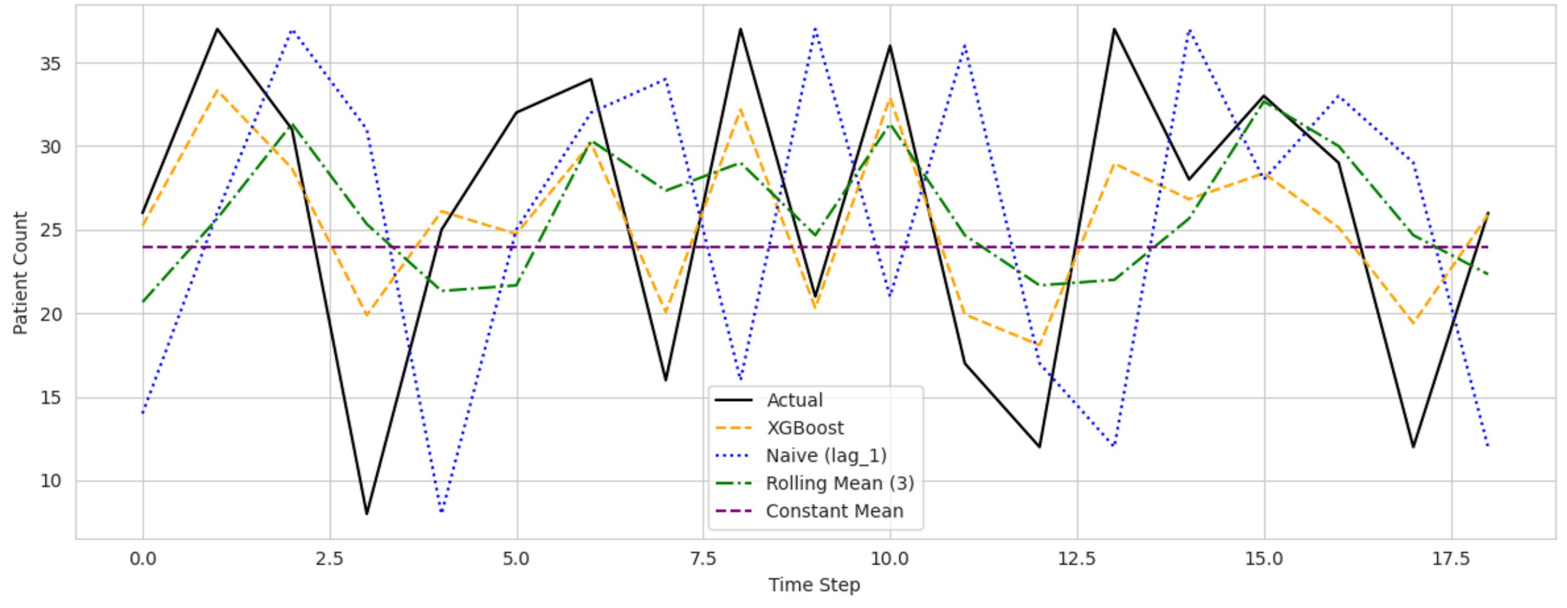
Predicted versus actual daily patient counts across Hospital ID 102 XGBoost = Extreme gradient boosting

**Figure 3:**
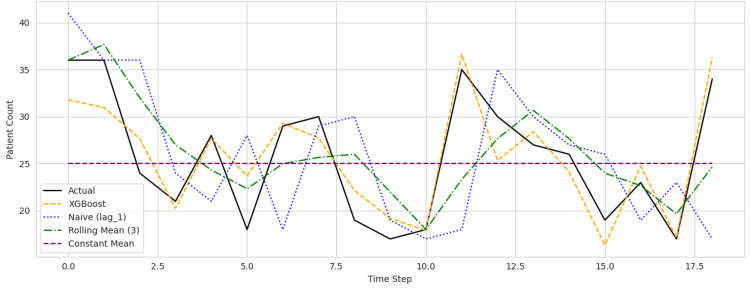
Predicted versus actual daily patient counts across Hospital ID 103 XGBoost = Extreme gradient boosting

As seen in Figures 1-3, prediction plots for each hospital revealed that the XGBoost model accurately followed the underlying trends in patient counts while reducing prediction volatility. Compared to the rolling mean and naive baselines, the model's forecasts were consistently closer to actual values, particularly during high-volume periods and sudden demand shifts.

Feature importance

**Figure 4:**
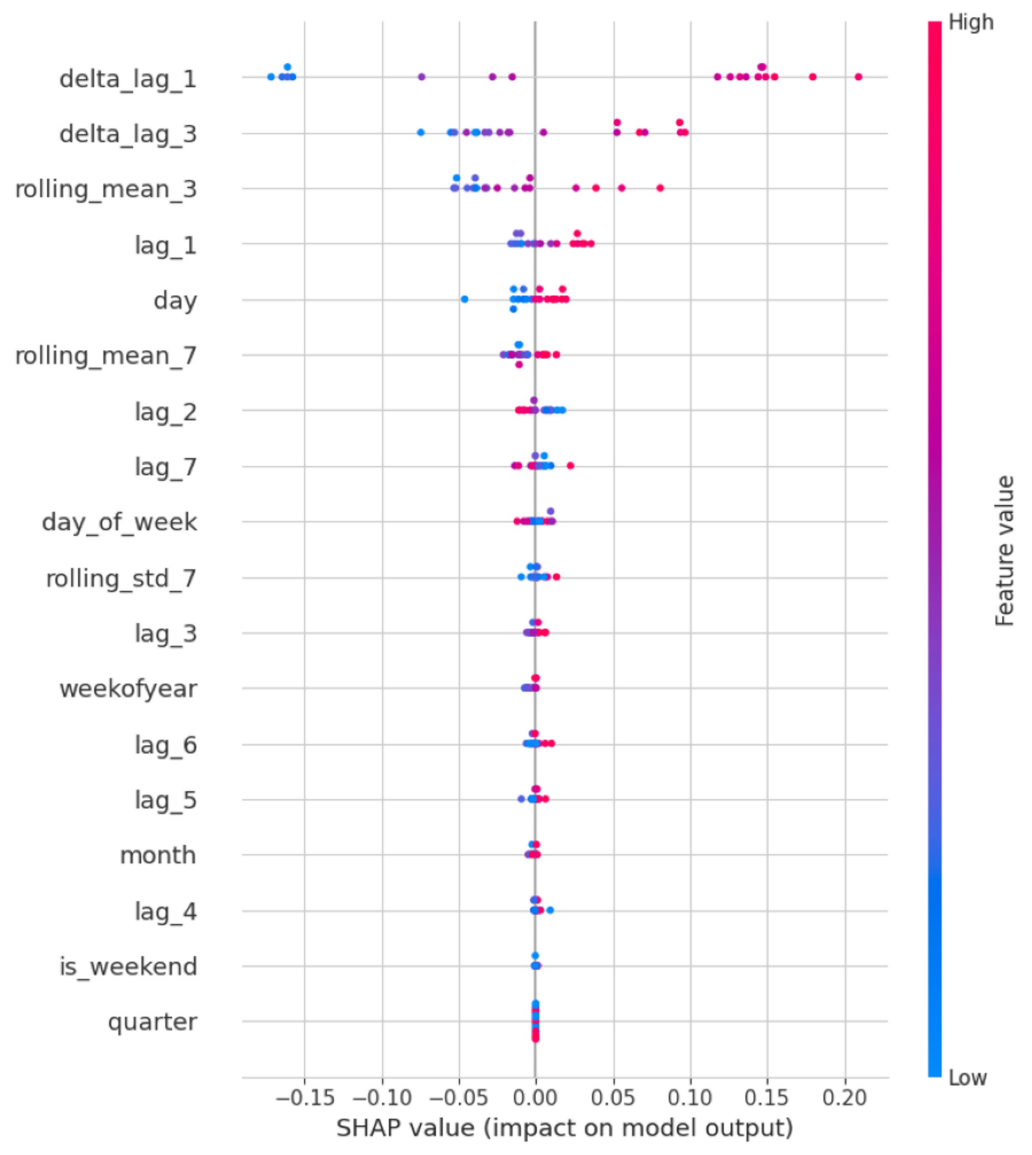
SHAP summary plot highlighting feature importance for Hospital ID 101 lag_1, lag_2, ..., lag_7 = Value from n days ago (e.g., lag_3 is the value from three days prior); delta_lag_1, delta_lag_3 = Change in value compared to n days ago (e.g., today’s value minus that of one or three days ago); rolling_mean_3, rolling_mean_7 = Moving average over the past n days (captures short-term trends); rolling_std_7 = Standard deviation over the past seven days (measures short-term variability); day = Day of the month (1–31); day_of_week = Day of the week (0 = Monday, 6 = Sunday); weekofyear = Calendar week number (1–52); month = Month of the year (1 = January, 12 = December); quarter = Quarter of the year (1–4); is_weekend = Binary indicator for weekends (1 = Saturday or Sunday, 0 = weekday)

**Figure 5:**
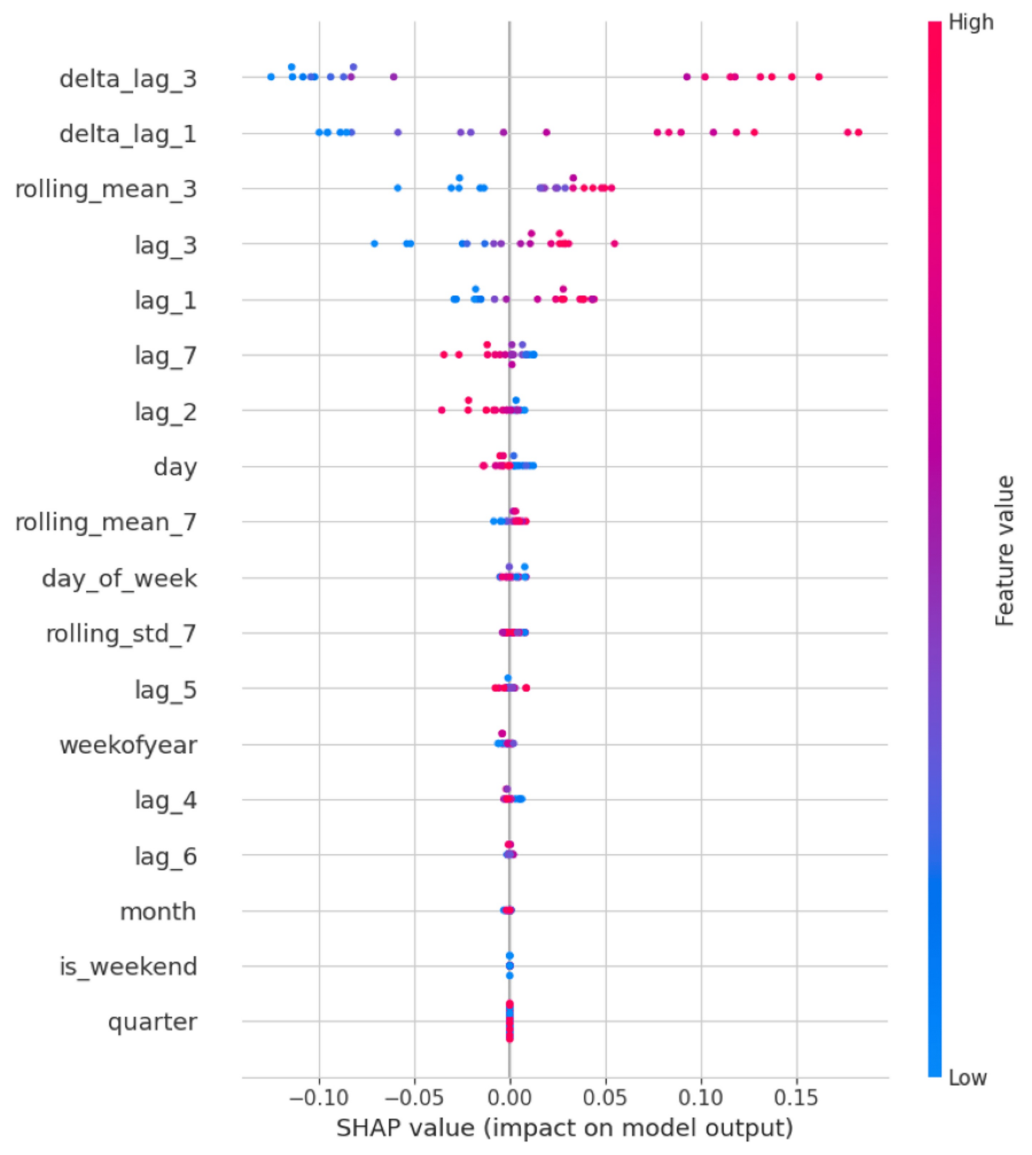
SHAP summary plot highlighting feature importance for Hospital ID 102 lag_1, lag_2, lag_3, lag_4, lag_5, lag_6, lag_7 = Values from n days ago (e.g., lag_3 = value from three days prior); delta_lag_1, delta_lag_3 = Day-over-day change compared to n days ago (e.g., today’s value minus value from one or three days ago); rolling_mean_3, rolling_mean_7 = Moving average over the past three or seven days (short-term trend smoothing); rolling_std_7 = Standard deviation of the past seven days (captures recent variability or fluctuations); day = Numeric day of the month (1–31); day_of_week = Day of the week (0 = Monday, 6 = Sunday); weekofyear = Week number of the calendar year (1–52); month = Month of the year (1 = January, 12 = December); quarter = Quarter of the year (1-4); is_weekend = Indicator for weekend days (1 = Saturday or Sunday, 0 = weekday)

**Figure 6:**
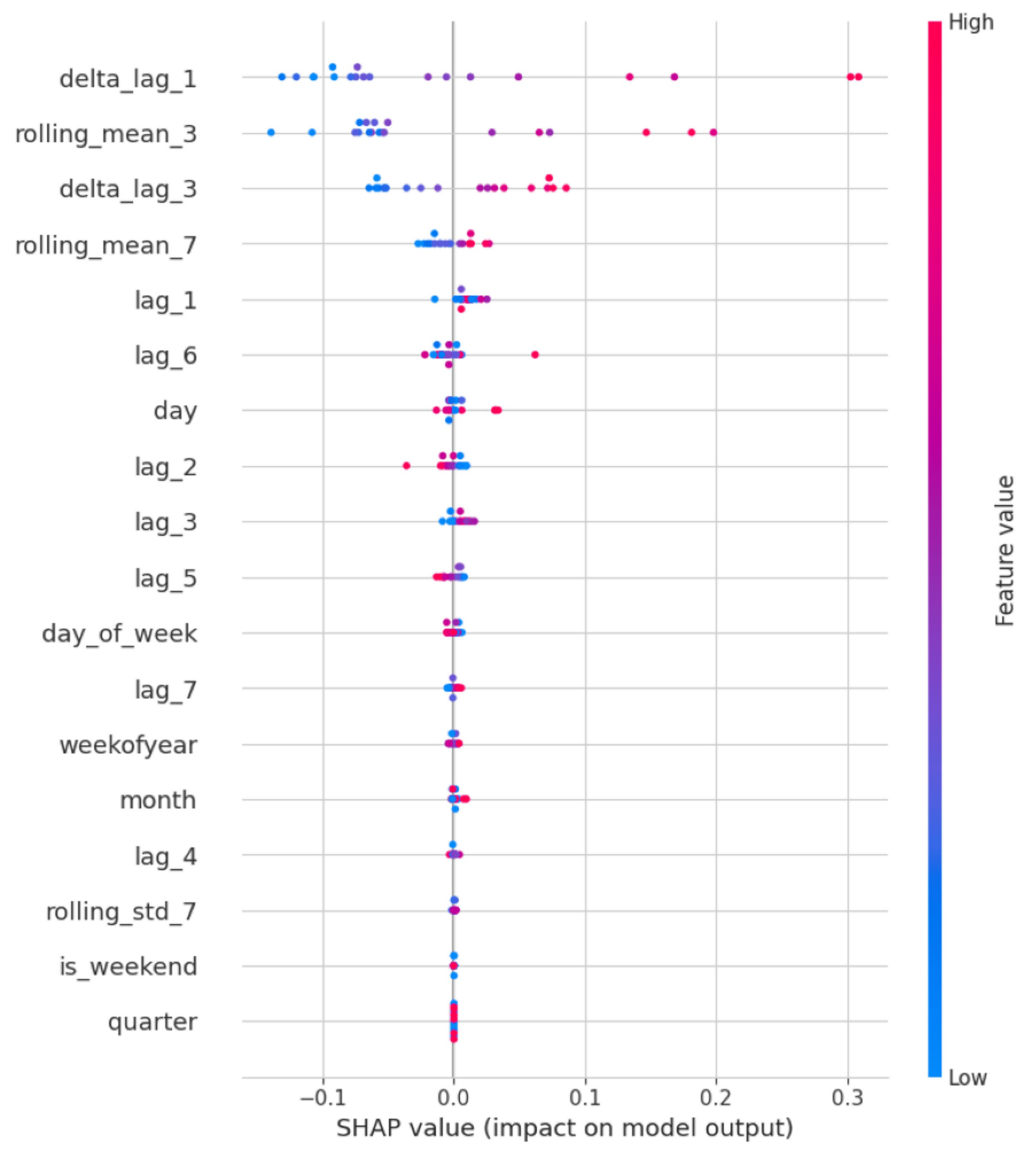
SHAP summary plot highlighting feature importance for Hospital ID 103 delta_lag_1, delta_lag_3 = Change in value compared to n days ago (e.g., today’s value minus that of one or three days ago); rolling_mean_3, rolling_mean_7 = Moving average over the past n days (captures short-term trends); lag_1, lag_2, ..., lag_7 = Value from n days ago (e.g., lag_3 is the value from three days prior); day = Day of the month (1–31); day_of_week = Day of the week (0 = Monday, 6 = Sunday); weekofyear = Calendar week number (1–52); month = Month of the year (1 = January, 12 = December); rolling_std_7 = Standard deviation of the past seven days (captures recent variability or fluctuations); is_weekend = Binary indicator for weekends (1 = Saturday or Sunday, 0 = weekday); quarter = Quarter of the year (1–4)

SHAP values provided insight into the model’s decision-making process. As illustrated in Figures 4-6, lag variables, especially lag_1, lag_2, and the three-day rolling mean, contributed most significantly to predictions across hospitals. Temporal features such as day of week also demonstrated consistent influence, particularly in hospitals with pronounced weekly visitation cycles.

## Discussion

Accurate forecasting of ED visit volumes is a longstanding challenge in healthcare operations management, particularly due to the inherent variability in patient inflow and the consequences of under- or over-resourcing. In this study, we developed and evaluated an XGBoost-based machine learning model that substantially outperformed several statistical baselines in predicting daily ER patient volumes across three simulated hospitals. The performance improvements observed, especially in terms of MSE, RMSE, and R², highlight the capability of gradient boosting methods to model nonlinear temporal patterns in hospital utilization effectively.

Across all three hospitals, the XGBoost model consistently surpassed naive, mean, and rolling average baselines. For example, in Hospital ID 103, the model achieved an R² of 0.81, compared to 0.36 for the rolling mean baseline, 0.00 for the mean baseline, and -0.58 for the naive lag-1 baseline. These results indicate that our model captured a significant signal in the temporal data that traditional approaches failed to exploit. Particularly, the inclusion of lag-based features (e.g., lag_1 through lag_7) and rolling averages (e.g., three-day mean) proved highly beneficial, as confirmed by SHAP value analyses, which demonstrated that recent historical patient volumes and day-of-week effects were among the most predictive variables. This aligns with existing literature suggesting that short-term lags and temporal patterns such as weekday-weekend effects are strong predictors of ER volumes [10,11].

One of the core strengths of this approach lies in its balance between model complexity and interpretability. Unlike deep learning models such as recurrent neural networks (RNNs) or long short-term memory (LSTM) networks, which are often criticized for their "black-box" nature, XGBoost models can be readily interpreted using SHAP values to understand feature contributions at both the global and instance levels [12]. This interpretability is especially important in healthcare contexts, where model transparency is often essential for stakeholder trust and regulatory compliance.

However, there are several limitations to consider. First, although the model performed well across the three hospitals, it was trained and evaluated on simulated datasets derived from a Kaggle source, which may not fully reflect the complexity and noise inherent in real-world hospital data. Additionally, some hospital datasets - such as Hospital ID 101 - appeared to have higher variance and fewer data points, which may have constrained model accuracy. This highlights a common limitation in healthcare machine learning: data sparsity and heterogeneity across institutions [13].

Another limitation stems from the reliance on recent historical patterns. While lag features are powerful in capturing momentum and short-term seasonality, they may struggle with unexpected events such as epidemics, extreme weather, or sudden policy changes, which were not explicitly modeled in this study. Moreover, temporal features such as public holidays, weather data, and local event calendars - known to affect ER visits - were not included in the current model. However, future work could integrate such exogenous variables to enhance generalizability and robustness [14,15].

From a methodological standpoint, this study also avoided cross-hospital learning due to the dataset structure. In practice, transfer learning or federated learning approaches could be used to share insights across hospitals while preserving data privacy - a relevant direction given the current emphasis on collaborative, privacy-conscious AI in healthcare [16].

Finally, considerations for deployment in real-time hospital settings must include automation of data ingestion pipelines, model retraining schedules, and user-friendly interfaces for healthcare staff. While XGBoost is computationally efficient and suitable for near real-time use, its adoption in clinical settings will require thoughtful integration with electronic health record (EHR) systems and operational workflows [17].

## Conclusions

XGBoost models enhanced with engineered temporal features demonstrate a marked improvement in forecasting emergency department volumes compared to traditional statistical approaches. While these results suggest strong potential for real-world generalization, it is important to note that findings are based on synthetic data, which may not fully capture the operational complexities and variability of real hospital environments. Additionally, key limitations include the absence of external predictors - such as weather, holidays, or public health alerts - and a reliance solely on historical utilization trends.

Future implementation steps should include validating the model on real-world, multi-institutional hospital data, integrating relevant external features, and testing performance in operational settings. The actionable insights generated by such models could inform decisions such as adjusting staff shift schedules, proactively preparing for anticipated surge days, and streamlining patient flow. To sustain forecasting accuracy, ongoing model monitoring and periodic retraining will be essential as hospital dynamics and patient patterns evolve over time.
